# Immunometabolic–uterine–ovarian interactions and flushing therapy in dairy cows: a narrative review

**DOI:** 10.14202/vetworld.2026.1097-1118

**Published:** 2026-03-17

**Authors:** Chandra Brahmantya, Aswin Rafif Khairullah, Imam Mustofa, Sri Mulyati, Tri Wahyu Suprayogi, Santoso Santoso, Erma Safitri, Saifur Rehman, Langgeng Priyanto, Bima Putra Pratama, Wasito Wasito, Riza Zainuddin Ahmad

**Affiliations:** 1 Doctoral Program of Veterinary Science, Faculty of Veterinary Medicine, Universitas Airlangga, Jl. Dr. Ir. H. Soekarno, Kampus C Mulyorejo, Surabaya 60115, East Java, Indonesia; 2 Research Center for Veterinary Science, National Research and Innovation Agency (BRIN), Jl. Raya Bogor Km. 46 Cibinong, Bogor 16911, West Java, Indonesia; 3 Department of Veterinary Reproduction, Faculty of Veterinary Medicine, Universitas Airlangga, Jl. Dr. Ir. H. Soekarno, Kampus C Mulyorejo, Surabaya 60115, East Java, Indonesia; 4 Research Center for Animal Husbandry, National Research and Innovation Agency (BRIN), Jl. Raya Bogor Km. 46 Cibinong, Bogor 16911, West Java, Indonesia; 5 Department of Pathobiology, Faculty of Veterinary and Animal Sciences, Gomal University, RV9W+GVJ, Indus HWY, Dera Ismail Khan 27000, Pakistan; 6 Department of Animal Science, Faculty of Agriculture, Universitas Sriwijaya, Jl. Masjid Al-Ghazali, Bukit Lama, Palembang 30128, South Sumatera, Indonesia; 7 Research Center for Process Technology, National Research and Innovation Agency (BRIN), KST BJ Habibie, Serpong, South Tangerang 15314, Banten, Indonesia

**Keywords:** biomarkers, dairy cows, immunometabolism, negative energy balance, ovarian hypofunction, reproductive efficiency, sustainable agricultural production, transition period, uterine flushing therapy

## Abstract

Ovarian hypofunction is a major reproductive disorder in dairy cows and contributes substantially to reduced fertility, prolonged days open, and economic losses. Increasing evidence indicates that this condition is not solely an ovarian problem but part of a broader immunometabolic disturbance that also affects uterine health during the postpartum transition period. Negative energy balance after calving leads to elevated circulating metabolites such as non-esterified fatty acids and β-hydroxybutyrate, which trigger oxidative stress and inflammatory signaling. These changes impair hypothalamic–pituitary–ovarian activity, suppress steroidogenesis, and delay follicular development. At the same time, metabolic stress weakens uterine immune defense, slows uterine involution, and increases susceptibility to endometritis, creating a reciprocal cycle in which uterine inflammation further inhibits ovarian reactivation. This narrative review synthesizes current knowledge on the bidirectional interactions between ovarian function and uterine health from an immunometabolic perspective. A structured literature search of major scientific databases was conducted, focusing on peer-reviewed studies addressing postpartum metabolism, immune responses, reproductive physiology, and non-hormonal therapeutic approaches in dairy cows. The review integrates endocrine, metabolic, and inflammatory mechanisms into a unified framework explaining how immune–metabolic imbalance disrupts reproductive homeostasis. Within this framework, uterine flushing therapy is discussed as a practical non-hormonal intervention aimed at restoring uterine conditions rather than directly inducing ovulation. By removing inflammatory exudates, reducing endotoxin load, improving endometrial perfusion, and supporting immune resolution, flushing may indirectly promote normalization of hormonal signaling and ovarian activity. Field evidence suggests that, when combined with appropriate nutritional and herd management strategies, this approach can improve pregnancy outcomes and reduce reliance on repeated hormonal treatments. Overall, adopting an immunometabolic management strategy that integrates metabolic monitoring, uterine health assessment, and targeted supportive interventions offers a promising pathway to enhance reproductive efficiency and sustainability in modern dairy production systems. Further standardized field trials and biomarker-guided protocols are needed to validate these approaches and facilitate their wider on-farm implementation.

## INTRODUCTION

Reproductive issues in dairy cattle directly impact production efficiency and farm profitability [1]. Conditions such as anestrus, repeat breeding, and delayed conception are especially prevalent during early lactation, when physiological and metabolic demands are highest [2]. Globally, ovarian hypofunction (OH) affects approximately 10%–30% of postpartum dairy cows, with higher prevalence in intensive production systems and during early lactation. Regional studies in developing dairy sectors report rates exceeding 35% under suboptimal nutritional and management conditions [3]. These disorders prolong the calving interval, reduce conception rates, and increase the costs of insemination and reproductive care, ultimately lowering the economic efficiency of dairy production systems [4].

OH is a major contributor to reproductive disorders in dairy cows, characterized by reduced ovarian activity that prevents dominant follicle development and normal ovulation [5]. This condition is closely associated with hormonal imbalance and metabolic disturbances during the transition period, when energy requirements rise sharply to support the onset of lactation [6]. During this phase, cows frequently experience negative energy balance (NEB), leading to excessive fat mobilization and increased circulating concentrations of non-esterified fatty acids (NEFA) and β-hydroxybutyrate (BHBA) [7, 8].

Beyond its direct effects on ovarian function, metabolic imbalance also has profound consequences for uterine health [9]. Reduced estrogen concentrations associated with OH decrease myometrial contractility and impair uterine defense mechanisms, increasing the risk of retained lochia, delayed uterine involution, and subclinical endometritis [10]. Economically, reproductive inefficiency linked to OH and uterine inflammation can result in losses ranging from tens to several hundred US dollars per affected cow per lactation, mainly due to extended days open, increased veterinary interventions, and reduced lifetime productivity [11, 12].

From a contemporary physiological perspective, immunometabolism provides a unifying framework for maintaining reproductive homeostasis by linking metabolic status with immune regulation [13]. Metabolic stress and postpartum inflammation can disrupt immune signaling in both the uterus and ovaries [14], making correction of this immune–metabolic imbalance essential for restoring reproductive function in dairy cows [15].

One non-hormonal strategy receiving increasing attention is uterine flushing therapy. This procedure involves flushing the uterine lumen with physiological or immunonutritional solutions to remove inflammatory exudates, improve endometrial perfusion, and modulate local immune responses [16]. Unlike conventional hormonal treatments that primarily induce ovulation, uterine flushing targets the uterine environment itself, with improvement in ovarian activity considered a secondary consequence of restored uterine–ovarian communication. This approach is particularly attractive for on-farm application because of its practicality and minimal interference with systemic hormonal balance [17].

Despite extensive research on postpartum reproductive disorders in dairy cows, most studies have examined OH, uterine disease, metabolic stress, and immune responses as separate entities. Existing reviews typically focus on endocrine control of ovarian activity, clinical aspects of endometritis, or nutritional management of transition cows, but rarely integrate these components into a unified physiological framework. In particular, the mechanistic links through which metabolic stress and immune activation simultaneously influence uterine recovery and ovarian reactivation remain insufficiently synthesized. Furthermore, while uterine flushing therapy has been reported as a practical non-hormonal intervention for improving uterine health, its role is often discussed primarily from a mechanical or clinical standpoint, with limited emphasis on its potential immunometabolic effects or its indirect influence on ovarian function. The lack of an integrative perspective combining metabolic biomarkers, immune signaling pathways, uterine–ovarian crosstalk, and supportive non-hormonal interventions represents a significant knowledge gap. Addressing this gap is essential for developing physiologically oriented and sustainable reproductive management strategies that move beyond symptom-based hormonal treatments toward mechanism-driven herd-level approaches.

Therefore, this narrative review aims to provide a comprehensive synthesis of current evidence on the bidirectional interactions between ovarian function and uterine health in dairy cows within an immunometabolic framework. Specifically, the review seeks to (i) summarize the endocrine, metabolic, and inflammatory mechanisms underlying postpartum OH and uterine dysfunction; (ii) elucidate how metabolic stress and immune dysregulation mediate crosstalk between the uterus and ovaries during the transition period; (iii) evaluate the biological rationale, physiological effects, and practical applications of uterine flushing therapy as a complementary non-hormonal intervention; and (iv) highlight the role of immunometabolic biomarkers and supportive management strategies in improving reproductive efficiency. By integrating these aspects, the review intends to present a coherent conceptual model linking metabolic status, immune regulation, and reproductive performance, thereby supporting evidence-based and sustainable approaches to fertility management in modern dairy production systems.

## REVIEW METHODOLOGY

### Study design

This study was conducted as a narrative review aimed at synthesizing current evidence on the immunometabolic links between OH and uterine health in dairy cows, with particular emphasis on non-hormonal flushing therapy as a complementary intervention. A narrative approach was selected to enable mechanistic integration of endocrine, immune, and metabolic pathways that are often examined separately in experimental and clinical studies.

### Literature search strategy

A structured literature search was performed using the PubMed, Scopus, and Web of Science databases. The search included peer-reviewed articles published between 2000 and December 2025. The following keywords and Boolean combinations were used: “ovarian hypofunction,” “postpartum anestrus,” “uterine health,” “endometritis,” “negative energy balance,” “immunometabolism,” “non-esterified fatty acids,” “β-hydroxybutyrate,” and “uterine flushing therapy.”

### Study selection criteria

The inclusion criteria were as follows: (i) original research articles, clinical trials, and relevant review papers; (ii) studies conducted in dairy cows; (iii) investigations evaluating ovarian function, uterine health, metabolic status, or immune responses during the postpartum period; and (iv) studies reporting outcomes related to reproductive performance or uterine recovery. Exclusion criteria included studies involving non-dairy species, non-postpartum animals, case reports lacking diagnostic criteria, and articles without relevance to reproductive physiology or immunometabolic mechanisms.

### Definitions and diagnostic standards

OH was defined as the absence of ovulation or dominant follicle development beyond 40–50 days postpartum, characterized by small inactive ovaries on ultrasonography (<8 mm follicles), low circulating progesterone concentrations (<1 ng/mL), and absence of a functional corpus luteum. Subclinical endometritis was defined based on cytological thresholds of ≥5–10% polymorphonuclear neutrophils (PMNs) at 21–35 days postpartum or ≥3–5% PMNs after 35 days postpartum in the absence of clinical signs. Uterine dysfunction included delayed involution beyond 45 days postpartum, persistent intrauterine fluid, or ultrasonographic evidence of inflammatory changes.

### Outcome measures

The effectiveness of uterine flushing therapy was evaluated using standardized reproductive outcomes, including pregnancy rate, interval to first estrus, days open, and indicators of uterine recovery, such as cytological and ultrasonographic improvement. Comparisons with hormonal or nutritional interventions were based on reported clinical outcomes and mechanistic relevance rather than formal meta-analysis.

### Selection and evaluation of immunometabolic biomarkers

Immunometabolic biomarkers were selected based on their documented association with postpartum metabolic stress, inflammation, and reproductive outcomes. These included metabolic indicators (NEFA, BHBA, glucose, and IGF-1), inflammatory markers (haptoglobin and ceruloplasmin), and cytokines (IL-1β, IL-6, IL-8, and TNF-α). Conflicting findings were interpreted in relation to differences in sampling time, parity, production system, and disease severity.

### Data synthesis approach

Evidence was synthesized using a thematic and mechanistic framework. Findings were classified into endocrine regulation, metabolic stress, immune activation, and therapeutic intervention domains. This structured synthesis enabled integration of bidirectional ovary–uterus interactions within an immunometabolic context.

## OVARIAN PHYSIOLOGY AND HORMONAL REGULATION

In dairy cows, ovarian function is regulated through coordinated interactions among the hypothalamic–pituitary–ovarian (HPO) axis, metabolic signals, and the animal’s physiological status [18]. The ovaries control estrous cyclicity, ovulation, and steroid hormone production, all of which are essential for fertility [19]. This section focuses on regulatory mechanisms relevant to postpartum ovarian recovery and dysfunction rather than providing a detailed description of estrous cycle phases.

Under normal postpartum conditions, ovarian cyclicity typically resumes within 20–40 days after calving, marked by dominant follicle emergence and first ovulation. In well-managed herds, regular luteal activity and estrous cycles are generally re-established by 40–60 days postpartum [20]. Delays beyond this period represent a significant deviation from normal physiology and are strongly associated with OH, NEB, and concurrent uterine inflammation, as discussed in later immunometabolic sections [21].

Follicle-stimulating hormone (FSH) initiates follicular development by stimulating a cohort of antral follicles [22]. Selection of the dominant follicle is accompanied by increased estradiol (E2) and inhibin secretion, which suppresses further FSH release [23]. Rising estradiol concentrations trigger the preovulatory luteinizing hormone (LH) surge, leading to ovulation and corpus luteum (CL) formation [24]. The CL secretes progesterone (P4), which supports luteal function and prepares the uterus for embryo implantation [25]. Physiologically, circulating progesterone concentrations exceed approximately 1 ng/mL during the functional luteal phase, whereas estradiol peaks during follicular dominance.

Postpartum OH is characterized by deviations from this physiological pattern, including persistently low estradiol concentrations, reduced LH pulse frequency, attenuated or absent LH surges, and delayed or insufficient progesterone secretion. These changes reflect impaired follicular growth and luteal insufficiency and frequently coexist with metabolic and inflammatory disturbances typical of early lactation [26].

Metabolic status plays a pivotal role in modulating ovarian responsiveness [27]. During early lactation, dairy cows commonly experience NEB due to increased energy demands for milk production [28]. This condition is associated with reduced circulating insulin and insulin-like growth factor-1 (IGF-1), both of which are critical for granulosa cell proliferation and estradiol synthesis [29]. Altered leptin signaling during NEB further compromises hypothalamic gonadotropin release, impairing follicular maturation [30]. These metabolic signals form a key link between nutrition and reproductive endocrinology and are further elaborated in Section 4 on immunometabolic ovary–uterus interactions.

Parity and genetic background also influence postpartum ovarian recovery. Compared with primiparous cows, multiparous high-producing cows often experience deeper and more prolonged NEB, increasing susceptibility to delayed ovulation. Likewise, high-yielding breeds such as Holstein–Friesian cattle are more vulnerable to metabolic stress–induced ovarian dysfunction than dual-purpose or indigenous breeds, which typically exhibit greater metabolic resilience [31].

Disruption of metabolic–endocrine balance ultimately leads to OH, defined by reduced follicular activity or failure to ovulate [32]. Oxidative stress and low-grade systemic inflammation, common during the postpartum period, play central roles in this process [33]. Excessive reactive oxygen species (ROS) production damages granulosa cells and suppresses FSH and LH receptor expression [34]. In parallel, proinflammatory cytokines such as TNF-α and IL-6 interfere with steroidogenesis and promote follicular atresia, linking immune activation to endocrine suppression [35]. Elevated NEFA and BHBA further exacerbate ovarian dysfunction through direct cytotoxic effects on oocytes and somatic ovarian cells, reinforcing the immunometabolic mechanisms discussed in subsequent sections [36].

## PATHOPHYSIOLOGY OF OH

OH is one of the most prevalent reproductive disorders affecting dairy cows during the early postpartum period [37]. Clinically, it manifests as prolonged anestrus and ovulation failure resulting from impaired endocrine signaling, metabolic imbalance, and ovarian follicular inactivity [38]. Rather than representing an isolated ovarian disorder, OH reflects a multisystem disturbance involving neuroendocrine regulation, energy metabolism, immune activation, and oxidative stress [39].

OH is typically diagnosed in cows that fail to resume ovarian cyclicity beyond 40–60 days postpartum in the absence of overt uterine pathology [40]. Practical assessment relies on repeated transrectal ultrasonography showing the absence of a functional CL, small or static follicles (<8 mm), and persistently low circulating progesterone concentrations (<1 ng/mL) [41, 42]. These indicators help differentiate OH from other postpartum reproductive conditions and align pathophysiological mechanisms with field-level diagnostic tools.

### Endocrine dysregulation of the HPO axis

OH can be classified into hypogonadotropic and normogonadotropic forms [43]. In the hypogonadotropic type, reduced gonadotropin-releasing hormone (GnRH) secretion from the hypothalamus decreases LH and FSH release from the anterior pituitary [44]. Consequently, follicular growth fails to reach dominance and ovulation does not occur [45].

In contrast, the normogonadotropic type is characterized by apparently normal circulating gonadotropin concentrations but diminished ovarian responsiveness, often due to altered LH/FSH receptor expression or impaired intracellular signaling within follicular cells [46]. This distinction is clinically important because both forms may show similar ultrasonographic findings but differ in responsiveness to hormonal or non-hormonal therapeutic interventions [47].

### Metabolic pathways and NEB

A central driver of postpartum OH is NEB associated with early lactation [48]. High energy demands for milk synthesis reduce circulating glucose, insulin, and IGF-1 levels, all of which are essential modulators of ovarian follicular growth. Reduced insulin and IGF-1 signaling suppress hypothalamic GnRH release and decrease LH pulse frequency, preventing follicles from reaching the ovulatory stage [49].

Blood metabolite profiling provides practical insight into these mechanisms [50]. Cows with OH commonly exhibit elevated NEFA and BHBA concentrations along with reduced glucose and cholesterol levels, the latter being a key precursor for steroid hormone synthesis [51, 52]. Excessive NEFA and BHBA exert lipotoxic effects on oocytes and granulosa cells, promoting apoptosis and further impairing ovarian activity [53].

### Inflammatory and oxidative stress mechanisms

Oxidative stress and low-grade systemic inflammation during the periparturient period contribute substantially to ovarian dysfunction [54]. Increased ROS production damages granulosa cells, reduces FSH receptor expression, and disrupts steroidogenesis [55]. Proinflammatory cytokines such as TNF-α and interleukin-6 (IL-6) also interfere with follicular development by suppressing aromatase activity, reducing estradiol synthesis, and impairing ovulation [56, 57]. These immunometabolic interactions provide a mechanistic bridge between metabolic imbalance and endocrine suppression, reinforcing the value of integrating metabolic and inflammatory biomarkers into reproductive assessment strategies.

### Morphological and management-related factors

Morphologically, ovaries affected by hypofunction are typically smaller, with smooth surfaces and absence of dominant follicles [43]. Developing follicles often undergo atresia characterized by granulosa cell degeneration, reduced vascularization, and follicular collapse [44]. The CL, when present, is usually inactive or regressing, resulting in inadequate progesterone production [45]. The resulting hypoestrogenic and hypoprogesteronic environment limits uterine stimulation and may delay postpartum endometrial regeneration [46].

Management-related stressors, including heat stress, overcrowding, inadequate ventilation, and high stocking density, can exacerbate NEB and elevate cortisol levels. These stressors further suppress reproductive hormone secretion and increase the risk of prolonged postpartum anestrus, particularly in high-producing dairy herds.

**Figure 1:**
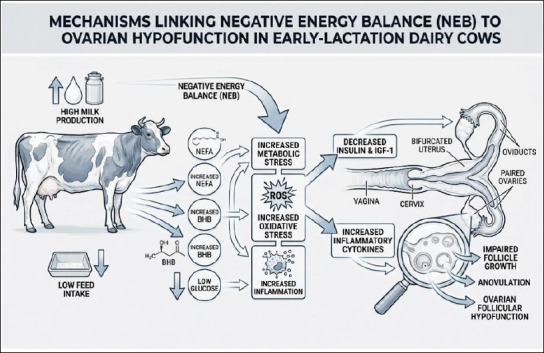
Pathways linking negative energy balance to ovarian hypofunction in early lactation dairy cows. This schematic illustration was generated using artificial intelligence tools (ChatGPT 5.2) and subsequently modified by the authors.

Figure 1 summarizes the integrated endocrine, metabolic, and inflammatory mechanisms underlying OH in early lactation dairy cows.

## IMMUNOMETABOLIC INTERACTIONS BETWEEN THE OVARY AND UTERUS

Immunometabolism refers to the integrated regulation of metabolic processes, immune function, and endocrine signaling in maintaining physiological homeostasis, including reproductive performance [58]. In dairy cows, immunometabolic balance serves as a central coordinating mechanism linking uterine recovery with ovarian reactivation during the postpartum transition period [59].

Immunometabolic interactions can be conceptualized as a sequential yet bidirectional process in which metabolic stress initially disrupts uterine immune homeostasis, whereas unresolved uterine inflammation subsequently feeds back to impair ovarian function through systemic inflammatory and endocrine signaling [60]. This framework provides a unifying synthesis of mechanisms that have often been described separately in previous reviews. Table 1 illustrates the reciprocal relationships among metabolic, immune, and hormonal systems in dairy cows during the transition period [61–78].

**Table 1:** Immunometabolic interactions between the ovary and uterus during the transition period in female dairy cows.

Components/Factors	Immunometabolic mechanisms	Impact on the uterus	Impact on the ovaries	Reproductive implications	References
Negative energy balance	Excessive adipose tissue mobilization elevates circulating NEFA and BHBA levels, which act as active metabolic signals that modulate immune cell function and activate NF-κB pathways	Induces oxidative stress and NF-κB activation in endometrial epithelial cells and macrophages, leading to increased production of IL-1β, IL-6, and TNF-α and delayed uterine immune homeostasis	Proinflammatory cytokines downregulate aromatase (CYP19A1) activity and FSH receptor expression in granulosa cells, suppressing estradiol synthesis	Impaired follicular dominance, delayed ovulation, and reduced luteal function	[61–65]

Postpartum uterine inflammation (endometritis)	Gram-negative bacterial infection releases lipopolysaccharide (LPS), which translocates systemically and activates TLR4-dependent inflammatory signaling	Sustains chronic endometrial inflammation and delays uterine involution and tissue repair	LPS–TLR4 interaction activates NF-κB and p38 mitogen-activated protein kinase pathways in granulosa and theca cells, suppressing steroidogenic acute regulatory and CYP19A1 expression and inhibiting steroidogenesis	Follicular atresia, ovulation failure, and prolonged anestrus	[66–70]

Activation of proinflammatory cytokines (TNF-α, IL-1β, and IL-6)	NF-κB-mediated cytokine overproduction links metabolic stress with immune and endocrine dysregulation	Increases vascular permeability and leukocyte infiltration, promoting chronic low-grade uterine inflammation	Direct suppression of ovarian steroidogenesis and exacerbation of ovarian hypofunction	The reproductive environment becomes unfavorable for conception	[63–65, 69]

AMPK–mTOR signaling axis	Imbalance between AMPK activation during energy deficiency and mTOR activation during inflammation or nutrient-excess amplifies immunometabolic dysregulation	Excessive mTOR activation promotes the synthesis of proinflammatory proteins and impairs endometrial regeneration	Reduced AMPK activity disrupts granulosa cell metabolism, survival, and follicular proliferation	Estrous cycle irregularities and delayed uterine recovery	[74–76]

Uterine microbiota dysbiosis	Postpartum dominance of pathogenic bacteria (e.g., *Escherichia coli*, *Trueperella pyogenes*) increases LPS burden and inflammatory tone	Sustains endometrial inflammation, disrupts mucosal immune tolerance, and inhibits uterine involution	Persistent systemic exposure to lipopolysaccharide and cytokines suppresses ovarian endocrine responsiveness	Reduced fertility and decreased pregnancy rates	[77, 78]

AMPK = AMP-activated protein kinase, BHBA = β-hydroxybutyrate, CYP19A1 = Aromatase, FSH = Follicle-stimulating hormone, IL-1β = Interleukin-1 beta, IL-6 = Interleukin-6, LPS = Lipopolysaccharide, mTOR = Mechanistic target of rapamycin, NEFA = Non-esterified fatty acids, NF-κB = Nuclear factor kappa B, p38 MAPK = p38 mitogen-activated protein kinase, TLR4 = Toll-like receptor 4, TNF-α = Tumor necrosis factor-alpha.

Excessive mobilization of adipose tissue during NEB results in elevated circulating concentrations of NEFA and BHBA [61]. These metabolites function not only as indicators of energy deficit but also as active metabolic triggers that directly modulate immune cell activity [62]. Prolonged exposure to elevated NEFA and BHBA concentrations induces oxidative stress and activates nuclear factor kappa B (NF-κB) signaling in macrophages and endometrial epithelial cells [63]. Consequently, NF-κB activation promotes overproduction of proinflammatory cytokines, including tumor necrosis factor-alpha (TNF-α), interleukin-1β (IL-1β), and IL-6 [64].

Although this inflammatory response is essential for postpartum uterine defense, sustained cytokine release exerts endocrine-disrupting effects that suppress ovarian steroidogenesis, primarily through downregulation of aromatase activity and FSH receptor expression in granulosa cells. This cause–effect cascade ultimately impairs follicular dominance, ovulation, and luteal function [65].

Uterine inflammation, manifesting as clinical or subclinical endometritis, further amplifies ovarian dysfunction through well-defined inflammatory crosstalk mechanisms [66]. Bacterial infection and retained postpartum debris promote the release of lipopolysaccharide (LPS) from Gram-negative bacteria [67]. Systemic translocation of LPS represents a critical mechanistic link, as LPS binds to Toll-like receptor 4 (TLR4) expressed on ovarian granulosa and theca cells, activating NF-κB and p38 mitogen-activated protein kinase pathways [68]. This sequence establishes a direct causal pathway in which uterine inflammation precedes and induces ovarian suppression rather than merely coexisting with it [69]. Furthermore, LPS inhibits expression of steroidogenic acute regulatory protein and cytochrome P450 aromatase (CYP19A1), reducing estradiol synthesis and preventing dominant follicle development [70].

Beyond NEFA and BHBA, additional immunometabolic mediators integrate metabolic status with uterine–ovarian signaling. Adiponectin, an adipokine whose concentration declines during NEB, enhances insulin sensitivity and supports ovarian steroidogenesis; thus, reduced adiponectin levels represent a mechanistic link between metabolic stress and increased inflammatory susceptibility [71]. Cortisol, elevated during metabolic or environmental stress, exerts dual effects by transiently suppressing uterine inflammation while chronically inhibiting gonadotropin secretion and ovarian responsiveness [72]. Acute-phase proteins such as haptoglobin and serum amyloid A serve as systemic biomarkers of immunometabolic imbalance and are consistently associated with delayed resumption of ovarian cyclicity and reduced fertility [73].

At the intracellular level, integration of metabolic and immune signals is mediated through the AMP-activated protein kinase (AMPK)– mechanistic target of rapamycin (mTOR) axis [74]. Activation of mTOR during inflammatory or nutrient-excess states promotes proinflammatory protein synthesis, whereas AMPK activation under energy-deficient conditions conserves cellular energy and restrains inflammatory signaling [75]. Disruption of the AMPK–mTOR balance in uterine and ovarian tissues compromises endometrial repair, granulosa cell survival, and ovulatory capacity, reinforcing a self-perpetuating immunometabolic loop between these organs [76].

The uterine microbiota further modulate these interactions. Under healthy conditions, commensal microbial communities support mucosal integrity and immune tolerance [77]. Postpartum dysbiosis, characterized by overgrowth of pathogenic bacteria such as *Escherichia coli* and *Trueperella pyogenes*, sustains chronic inflammation and increases systemic exposure to LPS, thereby intensifying OH through persistent immunometabolic signaling [78].

**Figure 2:**
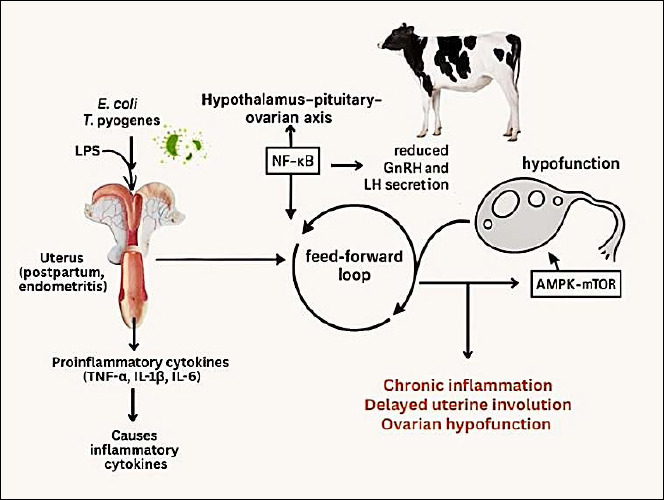
Bidirectional immunometabolic crosstalk between the uterus and ovary in dairy cows with endometritis. This schematic illustration was generated using artificial intelligence tools (ChatGPT 5.2) and subsequently modified by the authors.

An integrative schematic summarizing the cause–effect relationships among metabolic stress, immune activation, and endocrine disruption linking uterine inflammation and OH is presented in Figure 2 to enhance conceptual clarity.

## UTERINE HEALTH AND ITS RELATIONSHIP WITH OVARIAN RECOVERY

Uterine health in dairy cows is tightly regulated by ovarian endocrine activity and represents a critical determinant of subsequent ovarian recovery and fertility outcomes [79]. Deficits in estrogen and progesterone directly alter uterine immune competence, myometrial contractility, and endometrial regeneration when ovarian function is impaired, particularly in cases of OH, thereby creating an intrauterine environment that further suppresses ovarian reactivation [80, 81]. Thus, OH and uterine dysfunction should be regarded as functionally interdependent rather than isolated disorders.

In postpartum dairy cows, uterine health is routinely assessed using a combination of vaginal discharge scoring, transrectal ultrasonography, and endometrial cytology, all of which provide clinically actionable information relevant to ovarian outcomes. Clinical examination focuses on discharge characteristics, whereas ultrasonography enables evaluation of uterine involution, luminal fluid accumulation, and endometrial thickness—parameters strongly associated with delayed resumption of ovarian cyclicity [82]. Endometrial cytology obtained via cytobrush or low-volume uterine lavage allows detection of subclinical endometritis based on polymorphonuclear neutrophil (PMN) proportions, a diagnostic approach particularly relevant in cows with OH that lack overt clinical signs.

Histopathologically, the uterus of cows with OH exhibits bovine-specific postpartum alterations, including reduced endometrial epithelial thickness, glandular epithelium degeneration, and persistent leukocyte infiltration within the lamina propria [83]. Unlike cyclical endometrial remodeling in nonpregnant cows, postpartum endometrial repair is highly dependent on timely estrogen and progesterone signaling. Estrogen deficiency leads to endometrial atrophy and reduced uterine gland secretory activity, compromising the uterine milieu required for sperm transport and early embryonic survival [84]. Concurrently, insufficient progesterone impairs stromal cell proliferation and downregulates growth factors such as insulin-like growth factor-1 (IGF-1) and transforming growth factor-β (TGF-β), both essential for postpartum uterine repair and subsequent luteal support [85].

Clinically, endometritis severity, classified as mild, moderate, or severe based on discharge characteristics and PMN thresholds, correlates closely with ovarian functional outcomes. Increasing uterine inflammatory severity is consistently associated with prolonged postpartum anestrus, reduced dominant follicle emergence, delayed first ovulation, and diminished luteal progesterone concentrations, indicating a direct relationship between uterine pathology and OH.

From an immunological standpoint, estrogen and progesterone play species-specific regulatory roles in the bovine uterine immune system [86]. Estrogen enhances uterine defense by stimulating antimicrobial peptides (e.g., β-defensin and lactoferrin) and promoting controlled recruitment of neutrophils and macrophages into the uterine lumen [87]. In contrast, progesterone limits excessive inflammation by suppressing proinflammatory cytokines such as IL-1β and TNF-α while promoting anti-inflammatory mediators including IL-10 [88]. In dairy cows with OH, prolonged deficiency of these hormones leads to immune dysregulation characterized by impaired phagocytic activity, reduced mucus secretion, and persistent endometrial inflammation, all of which compromise fertility under commercial production conditions [89, 90].

Delayed uterine involution is a frequent clinical finding in cows with OH during the postpartum period [91]. Under normal physiological conditions, uterine involution is largely completed within 30–45 days postpartum, and ovarian cyclicity resumes with the first ovulation typically occurring around 20–35 days postpartum [92, 93]. Deviations beyond these time frames serve as practical clinical indicators of pathological uterine dysfunction [94, 95]. Low estrogen and progesterone concentrations reduce myometrial contractility, impairing clearance of lochia and placental remnants [96], thereby increasing susceptibility to secondary bacterial infection and perpetuating ovarian suppression [97].

The relationship between uterine dysfunction and OH is reciprocal and self-reinforcing [98, 99]. Chronic uterine inflammation suppresses hypothalamic gonadotropin-releasing hormone (GnRH) secretion, reduces LH pulsatility, and inhibits follicular maturation [100]. Conversely, ovarian hormone deficiency further compromises uterine immune competence and tissue regeneration [101]. This feed-forward loop highlights the clinical importance of prioritizing uterine recovery as a prerequisite for restoring ovarian cyclicity and improving conception outcomes [102].

Figure 2 illustrates the proposed mechanistic link between uterine inflammation, hypothalamic–pituitary–ovarian axis disruption, and OH.

## UTERINE FLUSHING THERAPY

Uterine flushing therapy is a non-hormonal intrauterine intervention primarily aimed at improving uterine health and facilitating postpartum uterine clearance in dairy cows, particularly in cases of mild to moderate endometritis and delayed uterine involution. Evidence-based studies consistently demonstrate improvements in uterine cleanliness and inflammatory status, whereas improvements in ovarian function should be interpreted as indirect secondary outcomes mediated through restoration of uterine–ovarian communication [103].

This approach involves intrauterine administration of sterile isotonic solutions, with or without adjunctive agents, to remove inflammatory exudate, reduce microbial load, and support endometrial repair [104].

### Basic principles of flushing therapy

Under physiological postpartum conditions, uterine clearance is achieved through coordinated myometrial contractions and leukocyte-mediated phagocytosis [105]. Reduced estrogen and progesterone concentrations impair these mechanisms in cows with OH, leading to fluid retention and prolonged inflammation [106].

Multiple clinical studies report that intrauterine lavage using isotonic saline (0.9% NaCl) or Ringer’s lactate facilitates physical removal of lochia, necrotic debris, and inflammatory fluid, thereby accelerating uterine involution [107].

Although no universally standardized protocol exists, most field studies describe the use of 500–2000 mL of sterile isotonic solution per treatment, administered once or repeated up to two or three times at 24–72-h intervals depending on clinical severity. Flushing is generally performed after 21–28 days postpartum, when acute metritis has resolved [108]. Warmed solutions (38°C–40°C) have been associated with enhanced uterine contractility and improved endometrial perfusion.

### Physiological and immunological effects

Beyond mechanical cleansing, uterine lavage increases endometrial microcirculation and stimulates myometrial activity, supporting faster involution [109]. Experimental and observational studies report enhanced leukocyte recruitment, particularly neutrophils and macrophages, following flushing [110].

At the mechanistic level, flushing therapy is proposed to modulate the local immune milieu, promoting a shift from persistent proinflammatory signaling toward resolution and tissue repair. Increased local expression of anti-inflammatory cytokines (e.g., IL-10 and TGF-β) has been reported, supporting epithelial regeneration [111].

Furthermore, reductions in intrauterine lipopolysaccharide (LPS) concentrations following lavage have been documented, which may attenuate LPS-mediated suppression of ovarian steroidogenesis via NF-κB signaling pathways [112, 113]. Although endocrine recovery is indirect, this provides a biologically plausible link between uterine flushing and gradual improvement in ovarian activity.

### Adjunctive flushing agents: immunonutrition and phytotherapy

Immunonutritional or phytotherapeutic additives have been incorporated into flushing solutions [114]. Minerals such as calcium (Ca), zinc (Zn), and selenium (Se) support antioxidant defenses and innate immune function within uterine tissue [115]. Through its role in glutathione peroxidase activity, selenium supplementation has been associated with reduced oxidative damage and improved uterine recovery [116].

Phytotherapeutic compounds, including Aloe vera, Curcuma longa, and Azadirachta indica, have demonstrated antimicrobial and anti-inflammatory properties in experimental and field studies [117]. Bioactive molecules such as curcumin and aloin reduce TNF-α and IL-1β expression while promoting fibroblast proliferation and endometrial repair [118].

These adjuncts should be considered supportive rather than standalone treatments, and their efficacy is maximized when combined with appropriate herd nutrition and management [119].

### Safety, contraindications, and procedural standardization

Uterine flushing should be performed using aseptic technique, sterile single-use catheters, and controlled infusion pressure. Potential risks include mechanical trauma, iatrogenic infection, and excessive uterine distension.

Flushing is contraindicated in cows with acute metritis, severe systemic illness, or confirmed pregnancy. Standardized biosecurity measures are essential to ensure animal welfare and reproducibility across clinical settings.

### Comparison with hormonal therapy

Hormonal therapies (GnRH, PGF_2_α, or combinations) remain standard tools for managing OH [120]. Although these protocols are effective in inducing ovulation, they do not directly address uterine inflammation or metabolic imbalance [121].

In contrast, uterine flushing directly targets uterine pathology. Field studies report pregnancy rates of approximately 35%–55% in cows with mild to moderate endometritis following flushing therapy, which is comparable to or slightly higher than outcomes achieved with hormonal therapy alone [122].

Additionally, reductions in days open of approximately 10–25 days have been documented, particularly when flushing is integrated with nutritional and management interventions [10, 103, 123]. From an economic standpoint, flushing therapy generally involves lower direct costs and reduced reliance on repeated hormonal treatments, making it especially relevant for small- to medium-scale dairy systems.

### Integrated and complementary use

Emerging evidence supports a sequential or combined strategy in which flushing therapy is applied to restore uterine health before hormonal synchronization. This approach improves the uterine environment prior to endocrine stimulation, thereby enhancing embryo survival [124].

Therefore, flushing therapy and hormonal protocols should be viewed as complementary, condition-based tools rather than competing interventions [125].

### Implications for ovarian function and reproductive performance

Restoration of uterine health through flushing therapy reduces inflammatory and endotoxin-mediated suppression of the hypothalamic–pituitary–ovarian (HPO) axis, facilitating normalization of GnRH and LH secretion [126]. Improved endometrial perfusion and oxygenation further support steroidogenesis and follicular development [127].

Thus, uterine flushing represents a non-hormonal, immunomodulatory intervention with indirect yet clinically meaningful benefits for ovarian recovery and overall reproductive efficiency [128].

### Immunometabolic perspectives in disease prevention and therapy

Reproductive health in dairy cows depends not only on hormonal balance but also on complex interactions between the immune system and metabolism, collectively termed immunometabolism [129]. In cases of OH, metabolic disturbances such as postpartum NEB play a major role in disrupting immune and endocrine homeostasis [130].

Immunometabolic strategies can be broadly categorized into (i) established nutritional and management-based practices currently applicable at the farm level and (ii) emerging or experimental interventions that require further validation. Decreased energy reserves and accumulation of metabolites such as NEFA and BHBA trigger oxidative stress and systemic inflammation, which in turn suppress ovarian function and worsen uterine conditions [131]. Accordingly, modern immunometabolic approaches emphasize feasible nutritional interventions and practical biomarker monitoring to prevent and manage reproductive disorders in a sustainable manner [132].

### Immunometabolic strategies with high practical feasibility

In dairy cows, the transition period represents a critical phase during which energy and protein requirements increase to support early lactation while feed intake typically decreases [133]. Prolonged NEB reduces circulating glucose, insulin, and insulin-like growth factor-1 (IGF-1) concentrations, which directly inhibit ovarian follicular development [134]. Therefore, the most established and widely adopted immunometabolic strategy for preventing OH is optimization of energy balance through appropriate ration formulation and targeted supplementation [135].

Supplementation with omega-3 polyunsaturated fatty acids (PUFAs), particularly eicosapentaenoic acid (EPA) and docosahexaenoic acid (DHA), is commonly administered at approximately 10–30 g/cow/day during the late dry period through early lactation. These fatty acids exert systemic anti-inflammatory effects by suppressing prostaglandin E_2_ (PGE_2_) and leukotriene B_2_ (LTB_2_) synthesis, thereby reducing uterine inflammation and supporting endometrial recovery [136].

Trace mineral supplementation, including selenium (0.3 mg/kg DM), zinc (40–60 mg/kg DM), and copper (10–15 mg/kg DM), is routinely implemented in commercial dairy systems and supports antioxidant defense mechanisms essential for ovarian and uterine recovery [137]. Similarly, vitamin E (1,000–3,000 IU/cow/day) and β-carotene (300–500 mg/cow/day) supplementation during the transition period has demonstrated consistent benefits under field conditions, including improved leukocyte function and faster resolution of postpartum uterine inflammation [138, 139].

### Emerging and experimental immunometabolic approaches

Several immunometabolic interventions remain experimental or are primarily supported by controlled or pilot studies. These include the use of specific cytokine biomarkers (e.g., IL-6 and IL-8), nutraceutical antioxidants, and advanced delivery systems such as nano-formulated supplements [140]. Although these approaches provide valuable mechanistic insights, their routine application is currently limited by cost, assay availability, and regulatory considerations [141].

Compounds such as resveratrol, curcumin, and N-acetylcysteine have shown potential to modulate NF-κB and mTOR signaling pathways and improve insulin sensitivity in reproductive tissues [142]. However, these compounds should be regarded as adjunctive or investigational until validated through large-scale field trials.

### Practical feasibility and on-farm implementation

Successful adoption of immunometabolic strategies depends on feasibility, cost-effectiveness, and ease of integration into existing herd management programs [143]. Although rumen-protected supplements offer improved bioavailability, their higher cost may limit their use in small- to medium-scale dairy operations [144, 145]. In contrast, monitoring metabolic indicators such as BHBA and NEFA using handheld devices is highly feasible at the farm level and allows early identification of cows at risk of OH [146].

Therefore, a tiered implementation strategy is recommended, prioritizing readily measurable metabolic indicators and established nutritional interventions while reserving laboratory-based biomarkers and experimental nutraceuticals for high-risk cows or research-focused herds.

### Integrated preventive framework

A multidisciplinary strategy encompassing nutritional management, uterine infection control, and supportive non-hormonal therapies is required for an effective immunometabolic-based preventive and therapeutic approach [103]. By prioritizing established, feasible interventions and selectively incorporating emerging approaches, immunometabolic management can realistically improve reproductive efficiency while minimizing reliance on hormonal treatments [147].

### Biomarker-based monitoring in immunometabolic reproductive management

This subsection focuses specifically on biomarker-based monitoring and its practical application to avoid conceptual overlap with earlier sections, rather than reiterating general immunometabolic mechanisms. Biomarker monitoring provides an objective framework for identifying cows at risk of OH and associated uterine disorders, particularly during the transition and early postpartum periods [148].

For editorial clarity and field applicability, immunometabolic biomarkers can be categorized into three functional groups: metabolic, inflammatory, and endocrine biomarkers, each serving a distinct diagnostic purpose.

### Metabolic biomarkers

Metabolic parameters are the most practical and widely used indicators for on-farm reproductive risk monitoring. Circulating concentrations of NEFA, BHBA, glucose, and IGF-1 reflect energy balance and metabolic stress during the transition period [149].

Plasma NEFA concentrations exceeding approximately 0.3–0.4 mmol/L during the late prepartum period and >0.6–0.7 mmol/L during the early postpartum period are commonly associated with an increased risk of OH and uterine disease [150]. Similarly, postpartum BHBA concentrations above 1.2–1.4 mmol/L indicate subclinical ketosis and impaired reproductive performance. Reduced IGF-1 concentrations during the first 2–4 weeks postpartum have been consistently linked to delayed ovulation and reduced dominant follicle growth [151].

From a practical standpoint, BHBA and, to a lesser extent, NEFA can be measured using handheld or semi-automated analyzers, making metabolic biomarkers highly feasible for routine herd-level screening.

### Inflammatory biomarkers

Inflammatory biomarkers provide insight into systemic and uterine immune activation but are generally better suited for targeted or confirmatory diagnostics than routine screening. Acute-phase proteins such as haptoglobin, ceruloplasmin, and albumin reflect the balance between inflammatory processes and nutritional status [152].

Haptoglobin concentrations below approximately 0.1 g/L are typically considered indicative of normal postpartum adaptation, whereas values exceeding 0.3–0.5 g/L during early lactation are frequently associated with subclinical inflammation, endometritis, and impaired luteal function. Elevated ceruloplasmin concentrations are likewise associated with uterine inflammatory disorders [153].

Cytokines such as IL-6 and IL-8 play key roles in local uterine immune responses. Although cytokine measurement is mainly confined to research settings, persistently elevated IL-8 concentrations in uterine fluid or serum may indicate sustained neutrophil recruitment and an unfavorable uterine environment for embryo implantation [154].

### Endocrine biomarkers

Endocrine biomarkers link metabolic and inflammatory status to ovarian function. Hormones such as IGF-1, insulin, progesterone, and estradiol provide complementary information on hypothalamic–pituitary–ovarian (HPO) axis activity [155].

Reduced IGF-1 and insulin concentrations during early lactation are associated with delayed follicular development and reflect prolonged NEB. Low circulating progesterone concentrations during the expected luteal phase may indicate impaired luteal function secondary to metabolic or inflammatory stress rather than primary ovarian failure [156].

### Temporal application and monitoring strategies

The diagnostic value of biomarkers is maximized when measurements are aligned with key physiological windows, including late prepartum (−21 to −7 days), early postpartum (0–14 days), and the peak reproductive risk period (14–35 days postpartum). Repeated measurements across these periods provide greater predictive accuracy than single-time-point sampling [157].

A tiered monitoring strategy is recommended to enhance practical applicability:(i) routine on-farm screening using metabolic biomarkers (BHBA and NEFA) for all transition cows, and(ii) targeted laboratory-based inflammatory or endocrine testing in cows identified as high-risk based on metabolic profiles or clinical findings [158].

### Integration into reproductive decision-making

When integrated with nutritional management and uterine health assessment, biomarker-based monitoring enables earlier and more targeted interventions, including nutritional adjustment, uterine flushing, or supportive non-hormonal therapies [149, 150]. This approach allows prevention or mitigation of OH before irreversible reproductive dysfunction occurs, thereby improving reproductive efficiency while reducing reliance on blanket hormonal treatments.

## FUTURE RESEARCH DIRECTIONS

Based on the key gaps identified in earlier sections, including limited field validation of immunometabolic biomarkers, lack of standardized non-hormonal intervention protocols, and challenges in translating experimental findings into herd-level practice, future research in immunometabolic-based reproductive management can be systematically organized into short-, medium-, and long-term priorities. This structured framework aims to facilitate progressive development from mechanistic understanding to practical implementation.

### Short-term research priorities: mechanistic validation and feasibility studies

In the short-term, research should focus on addressing the current lack of validated non-hormonal alternatives and biomarker-guided interventions identified in this review. Priority should be given to proof-of-concept and validation studies evaluating non-hormonal metabolic modulators capable of restoring immune–metabolic balance during the transition period [159].

Compounds such as resveratrol, curcumin, and N-acetylcysteine have demonstrated antioxidant and metabolic regulatory potential, including suppression of NF-κB and mTOR signaling pathways and improvement of insulin sensitivity and mitochondrial function [160]. However, their efficacy has largely been demonstrated under experimental or controlled conditions. Therefore, short-term studies should focus on dose optimization, safety evaluation, and biomarker-based assessment of reproductive responses, directly addressing the current gap in standardized efficacy metrics.

### Medium-term research priorities: translational optimization and protocol integration

Medium-term research should aim to bridge the translational gap between experimental findings and applied reproductive management. This includes optimization studies evaluating delivery systems, formulation stability, and interactions with conventional nutritional strategies [161].

The development of nanotechnology-based immunonutrition formulations represents a promising approach to overcome bioavailability limitations and inconsistent absorption highlighted earlier in the manuscript [162]. At this stage, uterine flushing therapy, immunometabolic supplementation, and precision biomarker monitoring should be integrated into standardized, condition-based reproductive protocols, allowing adaptive responses to metabolic stress and uterine inflammation.

### Long-term research priorities: system-level implementation and sustainability

Long-term research should address the current lack of large-scale, system-level evidence supporting immunometabolic reproductive strategies. Applied studies should focus on identifying specific molecular targets linking metabolic stress to ovarian and endometrial dysfunction and validating intervention strategies across diverse production systems [163].

Multicenter field trials evaluating reproductive performance, animal welfare, economic feasibility, and environmental sustainability should complement these efforts. Such studies are essential to confirm that immunometabolic approaches can achieve durable fertility improvements while reducing reliance on hormonal treatments, thereby supporting sustainable dairy production [164].

### Regulatory, ethical, and adoption considerations

In parallel with biological research, regulatory and adoption-related challenges identified earlier in the manuscript must be systematically addressed. Comprehensive toxicological and residue studies are required to ensure the long-term safety of non-hormonal metabolic modulators, particularly nano-formulated compounds, and to ensure compliance with food safety and animal welfare standards [165].

Regulatory approval pathways for novel feed additives and advanced formulations may vary across regions, potentially limiting widespread adoption. Additionally, practical constraints such as cost, ease of application, farmer awareness, and integration into existing herd management programs represent critical barriers to on-farm implementation [166, 167]. Addressing these challenges through interdisciplinary collaboration, stakeholder engagement, and extension-focused research will be essential for successful translation of immunometabolic innovations into routine dairy reproductive management [168].

**Figure 3:**
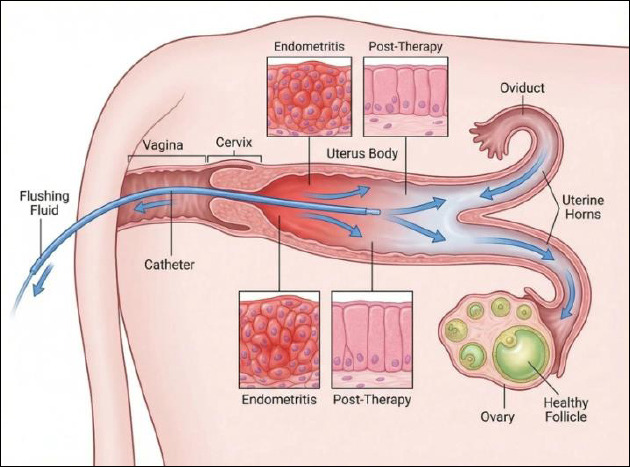
Intrauterine flushing therapy and ovarian recovery in dairy cows. This schematic illustration was generated using artificial intelligence tools (ChatGPT 5.2) and subsequently modified by the authors.

Figure 3 summarizes the proposed mechanistic framework linking postpartum uterine flushing, immunometabolic regulation, and restoration of ovarian function.

## CONCLUSION

This review highlights that OH in dairy cows is not an isolated ovarian disorder but a multifactorial condition arising from interconnected endocrine, metabolic, immune, and uterine processes. Evidence synthesized across physiological, clinical, and immunometabolic studies demonstrates that postpartum NEB, oxidative stress, and uterine inflammation act synergistically to disrupt hypothalamic–pituitary–ovarian signaling, impair follicular development, and delay the resumption of ovarian cyclicity. The bidirectional relationship between uterine health and ovarian function underscores the importance of considering reproductive disorders within an integrated immunometabolic framework rather than as separate pathological entities.

From a practical standpoint, this integrative perspective supports a shift toward mechanism-based reproductive management. Biomarker-guided monitoring of metabolic indicators such as NEFA, BHBA, and IGF-1 enables early identification of cows at risk, while nutritional optimization and transition period management remain foundational preventive strategies. Uterine flushing therapy emerges as a physiologically oriented, non-hormonal intervention that promotes uterine clearance, immune resolution, and endometrial repair, with indirect yet clinically meaningful benefits for ovarian recovery and fertility outcomes. When applied in combination with targeted metabolic support and reproductive monitoring, such approaches may reduce reliance on blanket hormonal treatments and improve herd-level reproductive efficiency.

A key strength of this review lies in its comprehensive integration of endocrine, metabolic, immune, and clinical evidence into a unified conceptual model linking uterine inflammation, immunometabolic imbalance, and ovarian dysfunction. However, limitations include reliance on heterogeneous experimental and field studies, variable biomarker thresholds across production systems, and limited large-scale validation of emerging non-hormonal interventions.

Future research should prioritize multicenter field trials, standardized biomarker-based protocols, and translational studies evaluating integrated management strategies under diverse dairy production conditions. Ultimately, adopting an immunometabolic-based approach that combines early risk detection, uterine health restoration, and supportive nutritional management offers a promising pathway to enhance reproductive performance, animal welfare, and sustainability in modern dairy production systems.

Overall, the evidence supports immunometabolic-based reproductive management as a realistic pathway to improve postpartum uterine recovery and accelerate ovarian reactivation while enhancing animal welfare and reducing dependence on repeated hormonal treatments. Implementing feasible on-farm screening (e.g., BHBA and NEFA), strengthening transition cow nutrition, and applying uterine-focused non-hormonal therapies in appropriately selected cows can collectively deliver more sustainable fertility outcomes in modern dairy production systems.

## DATA AVAILABILITY

All the generated data are included in the manuscript. The selection of articles and supplementary text can be made available from the corresponding author upon a request.

## AUTHOR’S CONTRIBUTIONS

CB, IM, and ARK: Drafted the manuscript. SM, TWS, and ES: Revised and edited the manuscript. SR, RZA, and LP: Participated in preparation and critically reviewed the manuscript. BPP, WW, and SS: Edited the references. All authors have read and approved the final version of the manuscript.
